# In Cellulo Evaluation of the Therapeutic Potential of NHC Platinum Compounds in Metastatic Cutaneous Melanoma

**DOI:** 10.3390/ijms21217826

**Published:** 2020-10-22

**Authors:** Elsa Charignon, Mathilde Bouché, Caroline Clave-Darcissac, Georges Dahm, Gabriel Ichim, Anthony Clotagatide, Hichem C. Mertani, Philippe Telouk, Julie Caramel, Jean-Jacques Diaz, Stéphane Bellemin-Laponnaz, Philippe Bouvet, Claire Billotey

**Affiliations:** 1 Hospices Civils de Lyon, EA3738-Therapeutic Targeting in Oncology, Université Jean Monnet-Université Claude Bernard Lyon1, 165 Chemin du Grand Revoyet, CEDEX, 69921 Oullins, France; elsa.charignon@laposte.net (E.C.); caroline.darcissac@gmail.com (C.C.-D.); anthony.clotagatide@chu-st-etienne.fr (A.C.); 2 INSERM 1052, CNRS 5286, Centre Léon Bérard, Centre de Recherche en Cancérologie de Lyon, Université de Lyon, Université Claude Bernard Lyon1, CEDEX 08, 69373 Lyon, France; hichem.mertani@lyon.unicancer.fr (H.C.M.); julie.caramel@lyon.unicancer.fr (J.C.); jeanjacques.diaz@lyon.unicancer.fr (J.-J.D.); philippe.bouvet@ens-lyon.fr (P.B.); 3 Institut de Physique et Chimie des Matériaux de Strasbourg, Université de Strasbourg-CNRS UMR7504, Strasbourg, Bâtiment 69, 23 Rue du Loess, 67200 Strasbourg, France; mathilde.bouche9@gmail.com (M.B.); georgesdahm@hotmail.com (G.D.); bellemin@unistra.fr (S.B.-L.); 4 Hôpital Nord, Département de Pharmacie, Centre Hospitalier Universitaire de Saint-Etienne, Avenue Albert Raimond, 42270 Saint-Priest, France; 5 Cancer Cell Death Laboratory, part of LabEx DEVweCAN, Cancer Initiation and Tumoral Cell Identity Department, Centre de Recherche en Cancérologie de Lyon, 69008 Lyon, France; gabriel.ichim@lyon.unicancer.fr; 6 Laboratoire de Géologie de Lyon Terre, Planètes, Université de Lyon, Environnement-ENS-UCBL-CNRS, UMR CNRS 5276 (CNRS, ENS, Université Lyon1), École Normale Supérieure de Lyon, 9 rue du Vercors, CEDEX 07, 69364 Lyon, France; philippe.telouk@ens-lyon.fr; 7 École Normale Supérieure de Lyon, Université de Lyon, 9 rue du Vercors, CEDEX 07, 69364 Lyon, France; 8 UFR de Médecine, Campus Santé Innovations, Université de Lyon, Université Jean Monnet, 10 rue de Marandière, 42270 Saint-Priest en Jarez, France

**Keywords:** platinum N-heterocyclic carbene complexes, metastatic cutaneous melanoma, chemotherapy, BRAF inhibitor resistance, apoptosis, chemoresistance

## Abstract

We describe here the evaluation of the cytotoxic efficacy of two platinum (II) complexes bearing an N-heterocyclic carbene (NHC) ligand, a pyridine ligand and bromide or iodide ligands on a panel of human metastatic cutaneous melanoma cell lines representing different genetic subsets including BRAF-inhibitor-resistant cell lines, namely A375, SK-MEL-28, MeWo, HMCB, A375-R, SK-MEL-5-R and 501MEL-R. Cisplatin and dacarbazine were also studied for comparison purposes. Remarkably, the iodine-labelled Pt-NHC complex strongly inhibited proliferation of all tested melanoma cells after 1-h exposure, likely due to its rapid uptake by melanoma cells. The mechanism of this inhibitory activity involves the formation of DNA double-strand breaks and apoptosis. Considering the intrinsic chemoresistance of metastatic melanoma cells of current systemic treatments, these findings are promising and could give research opportunities in the future to improve the prognosis of patients suffering from unresectable metastatic melanoma that are not eligible or that do not respond to the most effective drugs available to date, namely BRAF inhibitors and the anti-PD-1 monoclonal antibody (mAb).

## 1. Introduction

The incidence of cutaneous melanoma and other skin cancers has dramatically increased over the past five decades in Caucasian populations that are exposed to intense and frequent ultraviolet radiation [1], resulting in an estimated 100-fold difference in the rate of melanoma incidence between Australia and India in 2012 [2]. Thus, 80% of the 232,000 novel cases of melanoma estimated worldwide in 2012 and 65% of mortalities associated with melanoma occurred in Europe, North America and Oceania [2].

Indeed, approximately 70 to 90% of cutaneous melanoma are diagnosed at an early stage as primary tumors prior to metastasis [3,4] and are often cured following resection [5,6]. However, at the metastatic stage, cutaneous melanoma cells invade lymph nodes (known as stage IIIC for unresectable lymph node metastasis) and/or distant organs (stage IV), and require systemic procedures, except in the case of skin and/or lymph node metastases of limited sizes [7,8], with the aim of prolonging life and symptom-free survival beyond several months.

Recent insight into the therapeutic management of unresectable metastatic cutaneous melanoma came from, on one hand, the design of blocking monoclonal antibodies (mAbs) that enhance the antitumor activity of the immune system, and on the other hand, from small targeted molecules that inhibit the aberrant activation of mitogen-activated protein kinases (MAPKs) [9,10]. The molecular analysis of primary tumors or metastases should thus be performed to determine the innate BRAF-V600 mutational status and consequently patient eligibility for targeted therapy with BRAF inhibitors (BRAF = B-Raf proto-oncogene, serine/threonine kinase). Indeed, most metastatic cutaneous melanoma patients with mutated BRAF-V600 (around 40–50%) exhibit a spectacular and rapid initial response to the BRAF inhibitor treatments, which significantly enhances their overall survival (OS) and progression-free survival (PFS) compared to conventional chemotherapy, without resulting in a higher level of toxicity [2]. Nevertheless, many patients become secondarily resistant (i.e., their status reverts from “responder” to “non-responder”) upon treatment [3,6] and some develop important side effects among which is the onset of new primary BRAF wild type (BRAF-wt) cutaneous melanoma, likely due to the paradoxical activation of the MAPK pathway by BRAF inhibitors [5].

In innate BRAF-wt unresectable metastatic cutaneous melanoma, second generation mAbs blocking immune checkpoints, such as anti-PD-1, improve OS compared to conventional chemotherapies or anti-CTLA-4 [3,4]. Yet, roughly half of the patients do not respond to anti-PD-1, compromising their long-term survival [1], and there are currently no clinically-available biomarkers to predict patient response [11]. Several clinical trials in innate BRAF-m metastatic cutaneous melanoma demonstrated that the combination of either BRAF inhibitors with MEK inhibitors (MEK: Mitogen-activated protein kinase kinase) or of anti-PD1 with anti-CTLA-4 improve PFS compared to BRAF inhibitors or anti-CTLA-4 alone [9,10]. Hence, the development of new drugs aimed at significantly increasing patient OS and PFS remains a major challenge. Investigations focusing on the design and testing of new drugs that could be combined to small targeted molecules or immune checkpoint inhibitors without enhancing their toxicity are long awaited.

Transition metal complexes based on platinum (Pt), such as cisplatin and its derivatives carboplatin or oxaliplatin, induce apoptosis by forming DNA adducts and are efficiently used in the treatment of many types of solid tumors. However in addition to severe side-effects, resistance to such Pt compounds often appears over the course of treatment inducing a dose escalation, due to several mechanisms including the decrease in cellular accumulation of the metal salts resulting from a lower uptake and/or a higher efflux [12]. In the search for novel Pt-based drugs with possibly reduced side effects and resistance, various ligands were considered to stabilize Pt analogs. Among them, N-heterocyclic carbenes (NHCs) appear to be highly promising for the design of chemotherapies or antimicrobial agents, owing both to their versatile structures and stability [13,14]. Platinum complexes, such as [(NHC)PtX_2_(L)] (where L is a neutral nitrogen-based ligand and X a halogen), exhibit a high amount of DNA adducts in cisplatin-resistant cells [15,16]. They have been tested successfully on a variety of classical cancer cells, including cancer cells resistant to cisplatin [14,15,17]. Reactivity toward cutaneous melanoma (518A2 cells) has been investigated recently by Schobert et al. using bis-NHC Pt complexes [18]. Additionally, NHC gold(I) and platinum(II) complexes with picoline functionalized benzimidazolin-2-ylidene NHC ligands showed modest in vitro cytotoxic activities against murine melanoma cell line B16F10, which are highly metastatic [19]. 

**Figure 1:**
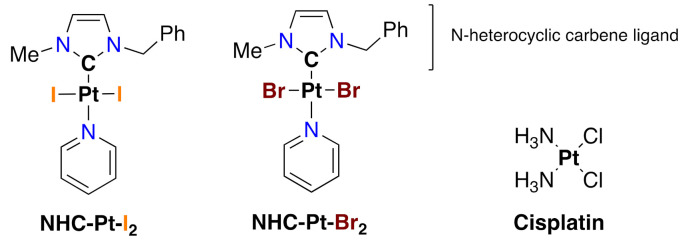
Molecular structure of the two N-heterocyclic carbene (NHC) platinum complexes, namely NHC-Pt-I_2_ (left) and NHC-Pt-Br_2_ (right) and cisplatin. The metal complexes are platinum (II) compounds stabilized by N-heterocyclic carbene and pyridine ligands positioned in a *trans* configuration (Ph = phenyl).

The purpose of the present study is to evaluate the cytotoxic efficacy of two promising NHC-Pt compounds, namely NHC-Pt-I_2_ and NHC-Pt-Br_2_ (Figure 1) compared to cisplatin and dacarbazine on 7 selected human cell lines. These cells lines were established from metastatic melanoma patient-derived tumor samples, including BRAF-inhibitor-resistant tumors, to represent the clinical heterogeneity of unresectable metastatic cutaneous melanoma both before and during treatment. To provide findings of clinical relevance and encourage the development of a treatment with a low level of toxicity, these cytotoxic effects were studied in cellulo after either a very short (1 h) or longer exposure (72 h). Based on our knowledge of the mode of action of cisplatin and of the pathways activated in tumor cells, we then investigated the interaction of these NHC-Pt compounds with cells, as well as the cell death pathways triggered by such an exposure.

## 2. Results

### 2.1. Effects of Pt Compounds on Various Cell Lines Viability

The cytostatic and cytotoxic effects of Pt compounds on viable cells were determined using an MTT assay with cells exposed to increasing concentrations of drugs to determine the half maximal inhibitory concentration (IC_50_) of each drug. To take into consideration the toxic effects of both first line and chronic treatment of metastatic cutaneous melanoma, this analysis was performed after a short-term drug exposure (1 h), as well as over a continuous period of 72 h.

#### 2.1.1. Effects Measured on Melanoma Cell Lines

Cell lines established initially from metastatic melanoma patient-derived tumor samples, either from skin tumors (A375, HMCB, SK-MEL-28/5) or lymph node metastasis (MeWo) were used to assess the anti-proliferative activities of NHC-Pt compounds in comparison with conventional single-drug chemotherapy, i.e., dacarbazine or cisplatin. The latter have been unsuccessfully proposed individually or in combination with other chemotherapies for metastatic melanoma. This panel encompassed two mutually exclusive genetic subsets of cutaneous melanoma, since MeWo and HMCB are BRAF-wt and NRAS mutated (NRAS-m), A375 and SK-MEL-28 are BRAF-m and NRAS wildtype (NRAS-wt).

While a clear difference could be observed after 72 h of treatment with cisplatin (Table 1) between BRAF-m/NRAS-wt and BRAF-wt/NRAS-m cells with a 10-fold lower IC_50_ for the former, only a relatively small difference was observed with NHC-Pt-I_2_ between these two groups. BRAF-m/NRAS-wt cells were thus more sensitive to cisplatin than to NHC-Pt-I_2_, though the latter displayed the greatest cytotoxic efficacy on BRAF-wt/NRAS-m cells. Dacarbazine and NHC-Pt-Br_2_ were exclusively efficient at limiting the proliferation of A375 cells. Hence, NHC-Pt-I_2_ had a cytotoxic activity on the four cell lines after 72 h of treatment.

Interestingly, when these cells were treated for only 1 h and the MTT assay was performed 72 h later (Table 2), NHC-Pt-I_2_ alone displayed cytotoxic effects towards the four metastatic cutaneous melanoma cell lines. Indeed, the IC_50_ for cisplatin, dacarbazine and NHC-Pt-Br_2_ could not be measured for most cell lines, after 1 h of treatment since high concentrations, are not sufficient to inhibit 50% of cell proliferation. In the case of A375 cells, while IC_50_ values for cisplatin and NHC-Pt-I_2_ were very similar after 72 h (1.6 μM vs. 2.5 μM, respectively), after only 1 h of treatment, cisplatin was less efficient (IC_50_: 24.6 μM), while NHC-Pt-I_2_ remained as cytotoxic (IC_50_: 2.3 μM).

Altogether, these data reveal that the IC_50_ for cisplatin and dacarbazine is very different depending on the cell line tested and on the duration of the treatment. In contrast, NHC-Pt-I_2_ was efficient irrespective of the treatment condition or cell line independently of their BRAF status, with the exception of the MeWo cell line, in which the IC_50_ after 1 h was twice that obtained after 72 h. Hence, NHC-Pt-I_2_ was the most cytotoxic compound.

#### 2.1.2. Impact on BRAF-Inhibitor-Resistant Cutaneous Melanoma Cell Lines

In order to include a very common clinical status of cutaneous metastatic melanoma observed in innate BRAF-m status patients treated for several months with BRAF inhibitors, we similarly assessed the cytotoxic efficacy of the 4 compounds on 3 generated vemurafenib-resistant cell lines, vemurafenib being an inhibitor of the BRAF enzyme used for the treatment of late-stage (IIIc and IV) melanoma (Table 3).

Dacarbazine was the least efficient drug in all cases, followed by NHC-Pt-Br_2_ (13.6 < IC_50_ < 25.3 μmol/L). Cisplatin displayed low IC_50_ values (1.0 to 6.2 μmol/L) when it was continuously present over 72 h. However, when cells were treated for only 1 h, cisplatin concentrations had to be increased 10-fold to observe similar IC_50_ values as the 72 h condition. Interestingly, NHC-Pt-I_2_ had a low IC_50_ in all three resistant cell lines, and these cytotoxicity values where comparable between cells treated for 1 h or 72 h (1.7 to 1.3 μmol/L), suggesting that the NHC-Pt-I_2_ compound could be a good cytotoxic agent to treat melanoma cell lines that have grown resistant to vemurafenib.

### 2.2. Investigation of Cell Pathways Involved in the Cytotoxicity of NHC-Pt Compounds

The cytotoxicity of platinum-based drugs arises from the activation of several cellular processes triggered by the formation of DNA adducts by Pt complexes located in the nucleus. Therefore, we studied the uptake and efflux and the impact of these compounds on DNA. Based on the results described above, highlighting the efficacy of NHC-Pt-I_2_ at inhibiting the proliferation of a large panel of human melanoma cell lines after only 1 h of exposure, our subsequent experiments focused exclusively on the A375 cell line exposed to the compounds for only 1 h.

#### 2.2.1. Evaluation of Pt Compound Uptake and Efflux Levels

The capacity of A375 cells to uptake Pt compounds was measured using ICP-MS in cell samples collected 1 h after the compound loading process, while compound efflux was measured similarly 24 h post-treatment by subtracting the amount of compounds within cells after 24 h from the initial amount. To avoid affecting the viability of cells treated with NHC-Pt-I_2_ over the time-course of the experiment, cells were exposed to a concentration of 1 μmol/L of each compound. The cell loading capacity of each compound and at each time-point was compared using a Mann Whitney test.

**Figure 2:**
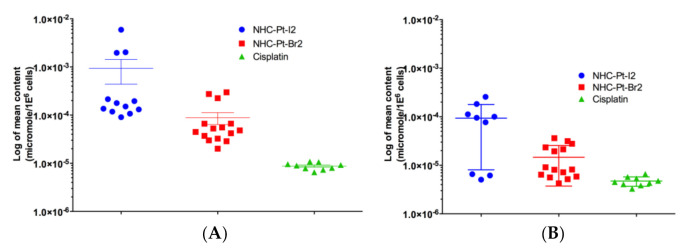
Uptake and efflux of Pt-based compounds. (**A**). Mean Pt cell content 1 h after the addition of the compound, represents uptake capacity of A375 cells measured in 9–15 samples per compound. (**B**). Mean Pt cell content 24 h after the addition of the drug, represents compound release or efflux measured in 9–15 samples per compound. Data are expressed in μmol per million cells as mean ± SEM.

The intracellular Pt concentration from NHC-Pt-I_2_ (9.36 × 10^–4^ ± 1.94 × 10^–3^ µmol/10^6^ cells, *n* = 12) after 1 h of incubation at a concentration of 1 μmol/L was statistically 11-fold higher (*p* = 0.014) than from NHC-Pt-Br_2_ (8.80 × 10^–5^ ± 9.38 × 10^–5^ µmol/10^6^ cells) and 107-fold higher (*p* < 0.0001) than from cisplatin (8.76 × 10^–6^ ± 1.46 × 10^–6^ µmol/10^6^ cells) (Figure 2A). It is well-known that iodine has a better affinity for platinum than bromine. Therefore, the formation of cationic Pt species in the presence of water will be increased in the case of bromide-containing complexes and these chemical interactions may have an impact on the overall cellular uptake of the platinum complexes. 

The mean intracellular Pt concentration starting from cisplatin at 24 h (4.73 × 10^–6^ ± 1.04 × 10^–6^ µmol/10^6^ cells) was significantly lower (*p* < 0.0001) than the initial amount loaded, while this difference was relatively smaller for NHC-Pt-Br_2_ (1.46 × 10^–5^ ± 1.089 × 10^–5^ µmol/10^6^ cells) with a lower statistical difference in compound cell content between 1 and 24 h (*p* = 0.0055) (Figure 2B). With NHC-Pt-I_2_, the mean intracellular Pt concentration at 24 h (9.35 × 10^–5^ ± 8.54 × 10^–5^ µmol/10^6^ cells) was not significantly different to the initial amount loaded (*p* = 0.162), and remained significantly higher (*p* = 0.0008) than with cisplatin, but not significantly different to that of NHC-Pt-Br_2_ (*p* = 0.0551). Of note, the level of NHC-Pt-I_2_ efflux may have been underestimated, as some of the released molecules could have been taken up once again by cells. In the case of cisplatin, its efflux would be enhanced by the saturation of the exporter proteins, and this phenomenon could be intensified with increasing compound concentrations.

#### 2.2.2. Evaluation of DNA Double-Strand Breaks

DNA double-strand breaks (DSB) are a major cause of cell death upon exposure to cisplatin [17]. DSB result in the phosphorylation of the variant histone H2AX at serine 139 (γ-H2AX). As the expression and recruitment of γH2AX could also be the consequence of apoptosis during DNA replication, it is important to determine the kinetics and the number of γH2AX foci.

**Figure 3:**
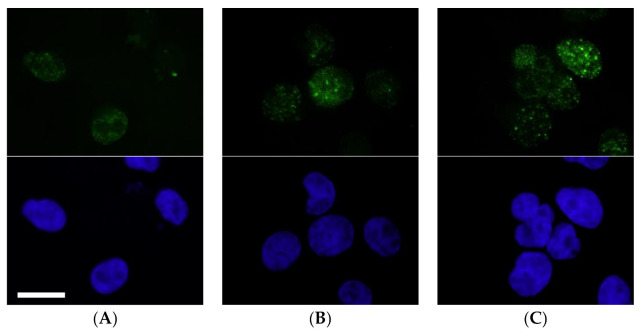
Qualitative monitoring of γH2AX foci formation triggered in A375 cells incubated with NHC-Pt compounds or cisplatin for 1 h. The green fluorescence corresponds to the detection of γ-H2AX and the blue fluorescence to nuclear DAPI staining. (**A**) correspond to γ-H2AX and DAPI staining captured 6 h post cisplatin treatment, (**B**) at 6 h post NHC-Pt-Br_2_ treatment, (**C**) at 6 h post NHC-Pt-I_2_ treatment (Scale bar = 10 µm).

As illustrated in Figure 3, at 6 h post-treatment, some γH2AX foci in cells treated with cisplatin or with NHC-Pt-Br_2_ were detectable. NHC-Pt-I_2_ was the compound inducing the most γH2AX foci. This rapid apparition of γH2AX foci in nuclei could suggest that DSB was directly induced by the NHC-Pt-I_2_ compound. 

#### 2.2.3. Assessing Cumulative DNA Damage

**Figure 4:**
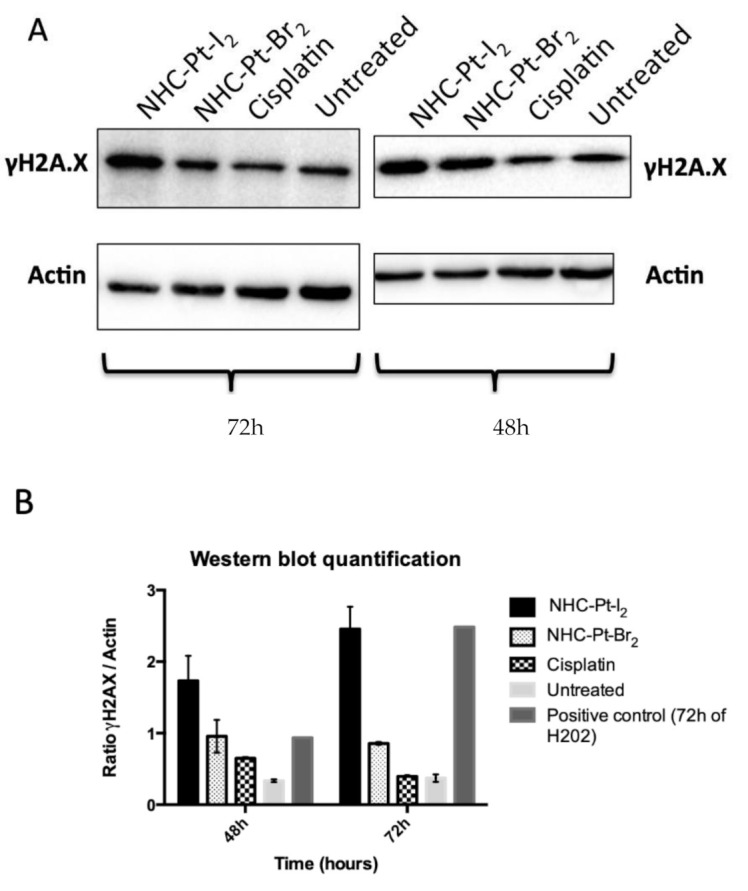
Cumulative expression of γ-H2AX resulting from 1 h of treatment of A375 cells with NHC-Pt compounds or cisplatin at their respective IC_50_ concentration. (**A**) Western blot analyses performed from cell lysates collected 48 h and 72 h after treatment. (**B**) Plot presenting the ratio of γ-H2AX expression to that of β-Actin (expressed as mean ± SD).

It is well-known that the cytotoxicity of cisplatin is modulated by the level of DNA damage and the result of several repair pathways [12]. Thus, we also investigated the level of cumulative DNA damage over a long period of time (48 h or 72 h) in cells treated with Pt compounds or cisplatin compared to untreated cells (Figure 4). The quantitative data from Western blot performed with cell lysates collected 48 h and 72 h after treatment enabled us to estimate cumulative DNA damage in cells. After 48 h post-treatment, the compound NHC-Pt-I_2_ induced a high level of DSB, followed by NHC-Pt-Br_2_. At 72 h post-treatment, NHC-Pt-I_2_ induced the most important level of DSB whereas the cisplatin impact was negligible compared to the untreated control cells. Additionally, we recently confirmed the ability of such NHC platinum complexes to interact with the DNA strands and revealed a highly compact and dense structure of Pt compounds bridging the DNA strands, while using optical tweezers and atomic force microscopy (AFM) [20,21].

#### 2.2.4. Cell Death Pathways

Cell death induced by cisplatin is mainly due to an apoptosis program similarly to other DNA-binding chemotherapies, although an excessive level of DNA damage could cause necrosis via a hyper-activation of poly-ADP-ribose polymerase [22,23].

Caspase-dependent death

**Figure 5:**
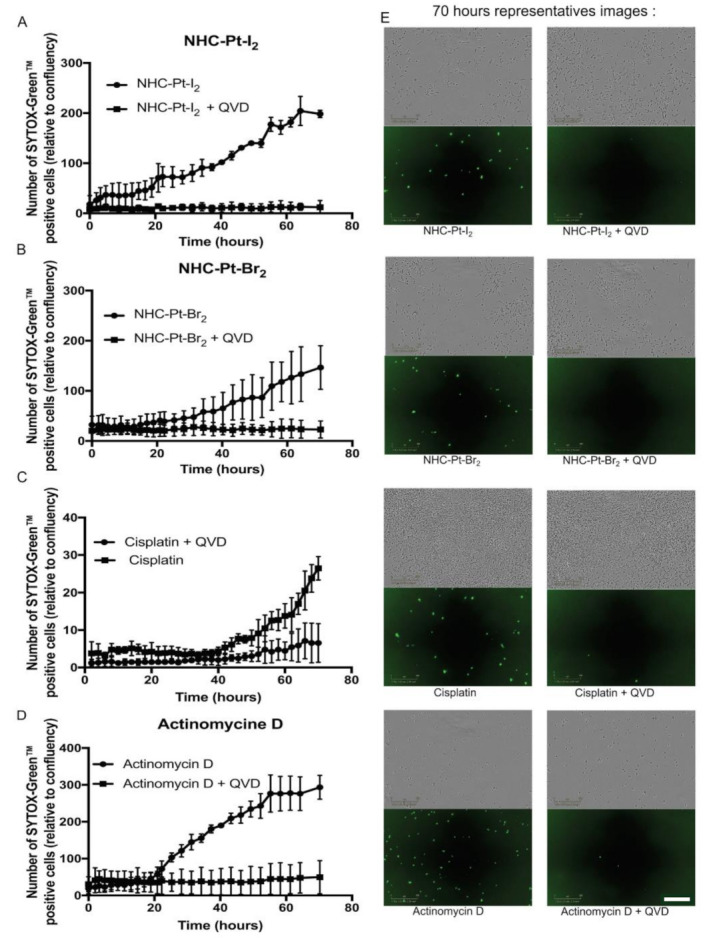
Evaluation of the role of caspases in NHC-Pt-induced cell death. A375 cells were incubated for 1 h treatment with (**A**) NHC-Pt-I_2_ (2.5 μmol/L), (**B**) NHC-Pt-Br_2_ (12 μmol/L), (**C**) cisplatin (2 μmol/L) or (**D**) Actinomycin D (30 μmol/L). Upon exposure, caspase activity was monitored by labelling cells with the SYTOX Green^™^ marker and culturing them for up to 70 h in the absence or the presence of the pan-caspase inhibitor Q-VD-OPh. Plots present the proportion of dead cells (mean ± SD, *n* = 3) calculated from 3 independent measures automatically taken every 30 min for 70 h with an IncuCyte^®^ live-cell imaging system. (**E**) Phase contrast and green fluorescence images acquired at 70 h for each condition (with Q-VD-OPh, right hand side or without, left-hand side). Scale bar (bottom right corner) = 300 µm.

Apoptosis results either directly from a transduction of DNA adduct signals via various pathways such as MAPK, or indirectly following mismatch repair processes via the activation of several cell-cycle regulators, such as P53. Irrespective of the initial trigger, the occurrence of apoptosis requires the activation of specific cysteine proteases of the caspase family. Thus, to investigate the underlying mechanisms of apoptosis, cell death was monitored in real-time following 1-h exposure of A375 cells to NHC-Pt compounds and cisplatin at their respective IC_50_ (i.e., 2.5 μmol/L for NHC-Pt-I_2_, 12 μmol/L for NHC-Pt-Br_2_ and 2 μmol/L for cisplatin) in the absence or in the presence of a pan-caspase inhibitor (Q-VD-OPh). These effects were compared to those of Actinomycin D, an intercalating DNA drug (Figure 5).

Data collected from three independent experiments were comparable and illustrated in Figure 5. As expected, Actinomycin D (Figure 5D) induced a linear SYTOX Green^TM^ uptake between 20 h and 70 h, whereas the presence of Q-VD-OPh prevented this uptake. In the case of cisplatin (Figure 5C), the presence of Q-VD-OPh protected the cells from cell death for up to 60 h, at which point a small proportion of cells (<10%) started taking up the SYTOX GreenTM. In the absence of Q-VD-OPh, the uptake of SYTOX Green^™^ began at 40 h and reached 25% at 70 h. For NHC-Pt-I_2_ and NHC-Pt-Br_2_ (Figure 5A,B) the presence of the pan-caspase inhibitor protected the cells from cell death, whereas its absence led to the linear uptake of SYTOX GreenTM from 10 h (NHC-Pt-I_2_) and 35 h (NHC-Pt-Br_2_) onwards for up to 55–60 h.

These results demonstrate that the death of cells treated for 1 h with NHC-Pt-I_2_ or NHC-Pt-Br_2_ was caspase-dependent, suggesting a major role of apoptosis in the cytotoxicity of these compounds. However the added contribution of a secondary necrosis resulting from an unfinished apoptotic program is also possible.

Cell death and the overexpression of Bcl-xL

The key role of resistance to apoptosis of melanoma cells in the chemoresistance of metastatic cutaneous melanoma was clearly demonstrated [24] to be related to the overexpression of the anti-apoptotic Bcl-xL protein [25]. Bcl-xL is a protein located on the mitochondria and acts as an anti-apoptotic protein by preventing mitochondrial permeabilization and ultimately lethal caspase activation.

**Figure 6:**
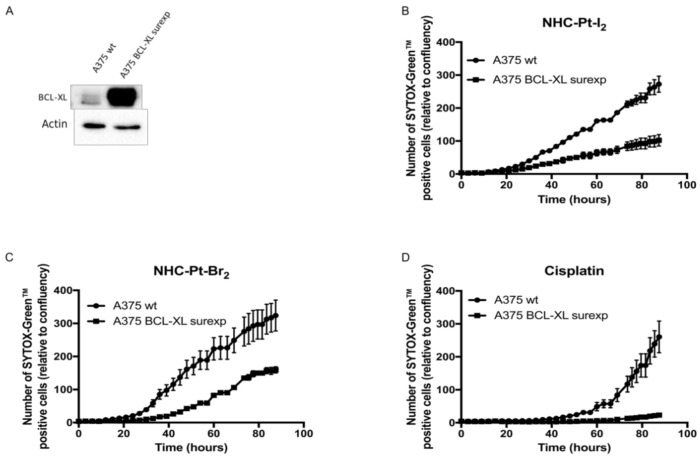
Impact of Bcl-xL overexpression on the cytotoxic efficacy of Pt compounds. (**A**) The level of expression of Bcl-xL by A375 cells was controlled by Western blot. (**B–D**) Standard A375 cells or Bcl-xL-overexpressing cells were treated for 1 h with (**C**) NHC-Pt-Br2, (**B**) NHC-Pt-I2, or (**D**) cisplatin at their respective IC_50_, then labelled with a specific dye to stain dead cells (SYTOX GreenTM). Data represent the proportion of dead cells (mean ± SD) calculated from 3 independent measures taken automatically every 30 min over 83 h with an IncuCyte^®^ live-cell imaging system.

To determine whether the overexpression of Bcl-xL affects the cytotoxic efficacy of NHC compounds, we monitored in real-time the death of both standard A375 cells or A375 cells that overexpress Bcl-xL after incubation with NHC-Pt-compounds and cisplatin at their respective IC_50_ for 1 h. The overexpression of Bcl-xL by A375 cells was validated by Western blot analysis (Figure 6A). Cell death was measured through the uptake of SYTOX GreenTM, a cell death marker. The level of SYTOX Green^™^ uptake by cells was quantified in real time (IncuCyte^®^). Cisplatin induced an uptake of SYTOX GreenTM in A375 after 50 h of treatment, while A375 cells overexpressing Bcl-xL displayed a negligible uptake of SYTOX GreenTM, indicating that cisplatin-induced cell death was efficiently blocked by Bcl-xL (Figure 6D). NHC-Pt-I_2_ and NHC-Pt-Br_2_ (Figure 6B,C) triggered a linear uptake of SYTOX GreenTM in A375 only 20 h after treatment, showing that these NHC-Pt compounds induce a more rapid cell death compared to cisplatin. In cells overexpressing Bcl-xL, cell death was drastically reduced but was not completely abolished as observed in the case of cisplatin, suggesting that apoptosis by Bcl-xL was not the only mechanism implicated in cell death induced by NHC-Pt compounds.

## 3. Discussion

The intrinsic aggressiveness of metastasis melanoma cells that results in their resistance to chemotherapy has been known for 20 years. Furthermore, several clinical trials have revealed that dacarbazine, which was until recently the sole FDA-approved drug for stage IIIc and IV melanoma, provides a very low (5–12%) objective rate of remission, which is defined as a decrease in the tumor mass of over 50% [7].

Here, we unveiled the drastic effect of NHC-Pt-I_2_, an NHC-containing platinum complex, in strongly inhibiting the proliferation of a large panel of human melanoma cell lines. This metabolic effect was due to its elevated and rapid uptake by cells, leading to the formation of abundant DNA strand breaks and apoptosis. Remarkably, the efficacy of NHC-Pt-I_2_ was far greater than cisplatin and dacarbazine after only 1 h of exposure, as evidenced by its very low IC_50_ in the low micromolar range. Although its cytotoxicity was lower towards the cell line derived from a metastatic site (MeWo) compared to the three cell lines derived from primary tumors, NHC-Pt-I_2_ displayed significant cytotoxicity compared to other tested compounds.

The duration of compound exposure (1 h) was selected to mimic clinical chemotherapy protocols that are delivered intravenously over 15–30 min or 2 h and rapidly cleared from the bloodstream within 1.38 h ± 0.9 h or 0.5–3.5 h in the case of dacarbazine and cisplatin, respectively [26]. Our findings demonstrate a higher anti-proliferative efficacy of cisplatin over dacarbazine in all of the cell lines, that is consistent with the fact that cisplatin is one of the most active antitumor agents used in human chemotherapy. Indeed, cisplatin which is delivered at a two-fold lower dose than dacarbazine due to its higher toxicity, provides a similar objective rate of remission (≤10%) as dacarbazine [26], hence confirming the potential of Pt based chemotherapeutics in the management of metastatic cutaneous melanoma.

Mechanistically, we demonstrated that the cytotoxic effect triggered by the exposure of A375 cells to the NHC-Pt-I_2_ complex for 1 h led to a caspase-dependent apoptosis. Interestingly, A375 cells overexpressing the anti-apoptotic protein Bcl-xL were also affected by NHC-Pt-I_2_, although they displayed a lower rate of apoptosis. Further mechanistic studies are required to determine the other pathway of cell death induced by NHC-Pt compounds and investigate the impact of mutated p53, the role of which has been established in the resistance to chemotherapy, particularly to cisplatin [24,26].

Additionally, the higher cellular uptake of NHC-Pt-I_2_ suggests the participation of a passive cell entrance flux in contrast with cisplatin which mainly enters cells via the copper-transporting P-type adenosine triphosphate. Therefore, NHC-Pt-I_2_ uptake may not be affected by the mutation of the CTR1 copper transporters associated with resistance to cisplatin in many types of cancers.

Aberrant activation of Raf/Mek/ERK MAPK is known to be strongly implicated in the proliferation and survival of cutaneous melanoma cells, by increasing the phosphorylation of downstream targets which results in excessive cell survival and proliferation, independently of growth factors [24]. We chose to investigate the efficacy of NHC-Pt-I_2_ using clinically relevant genetic subsets of the metastatic cutaneous melanoma, namely NRAS-m and BRAF-m cell lines, since they represent 65% of the patients suffering metastatic cutaneous melanoma [25]. Moreover, constraints due to the long-term use of BRAF inhibitors, such as increased resistance, were also addressed by performing the compound tests with three cell lines rendered resistant to vemurafenib.

Future studies could also take into consideration NF-1 mutations, which are identified in roughly 10% of metastatic cutaneous melanoma [26] or other genomic markers, the predictive role of which, in the management of stage IV melanoma, may arise in the future.

The high level of plasticity and heterogeneity of metastatic cutaneous melanoma cells, as illustrated by the pathways providing the BRAF-i resistance of the innate BRAF-m cells, suggest that the successful treatment of this disease requires the combination of several therapies targeting different oncogenic pathways [27,28,29,30].

Based on the IC_50_ obtained for NHC-Pt-I_2_ after 1 h exposure of BRAF-m or BRAF-wt skin or lymph node metastatic cutaneous melanoma cells, we believe that NHC-Pt-I_2_-containing chemotherapies delivered at a low dose would be an interesting approach. Hence, we could speculate that co-delivery of NHC-Pt-I_2_ and BRAF inhibitors could improve the prognosis of unresectable metastatic cutaneous melanoma by delaying or preventing the onset of a resistance to BRAF inhibitors. It is worth noting that NHC-Pt-I_2_ could become a major therapeutic resource in BRAF-wt patients that do not respond to the anti-PD-1 mAb, as well as in BRAF-m patients following unsuccessful BRAF-inhibitor treatment.

## 4. Materials and Methods

### 4.1. Compounds

#### 4.1.1. Synthesis and Characterization of NHC-Pt Compounds

The NHC-Pt-I_2_ complex was synthesized according to our previous protocol [16,21,31]. The same procedure was used and modified for the synthesis of the NHC-Pt-Br_2_ complex, NaI being replaced by NaBr. We obtained a 52% isolated yield. ^1^H-NMR (CDCl_3_, 300 MHz, 20 °C): δ 4.11 (s, 3H, N-CH_3_), 5.83 (s, 2H, N-CH_2_), 6.64 (d, J = 2.1 Hz, 1H, CH=), 6.83 (d, J = 2.1Hz, 1H, CH=), 7.27–7.53 (m, 7H, 5H_Ar_ and 2H_Pyr_), 7.76 (tt, J = 7.6 and 1.6 Hz, 1H, H_Pyr_), 9.04 (dt, J = 5.0 and 1.6 Hz, 2H, 2H_Pyr_). ^13^C-NMR (CDCl_3_, 75 MHz, 20 °C): δ 37.9 (N-CH_3_), 54.2 (N-CH_2_), 120.0 (CH=), 122.4 (CH=), 124.9 (C_Pyr_), 128.2 (CH_Ar_), 128.8 (CH_Ar_), 128.8 (CH_Ar_), 135.7 (C_Ar_), 137.7 (C_Pyr_), 138.2 (C-Pt), 152.6 (C_Pyr_). The purity of both compounds was confirmed by elemental analyses. The molecular structures of the NHC-Pt-I_2_ and NHC-Pt-Br_2_ complexes are presented in Figure 1.

#### 4.1.2. Solution Preparation

The NHC-Pt compounds were diluted in water with a maximum of 0.9% of DMSO in the final solution. Cisplatin (MYLAN, Saint-Priest, France) and dacarbazine (MEDAC, Lyon, France) solutions were prepared according to the manufacturer’s recommendations and diluted at the appropriate concentration using the dedicated cell culture medium.

### 4.2. Cell Lines, Cell Culture, Treatments

#### 4.2.1. Cell Lines and Cell Culture

Four commercial cell lines which were initially established from metastatic melanoma patient-derived tumor samples, either from skin tumor (A375, HMCB, SK-MEL-28/5 and 501MEL) or lymph node metastasis (MeWo) were used to assess the anti-proliferative activities of NHC-Pt compounds. This panel included genetic subsets exhibited by patients with metastatic cutaneous melanoma, since A375 and SK-MEL-28 are BRAF-m and NRAS wildtype (NRAS-wt) represent approximately 50% of these patients, and MeWo and HMCB are BRAF-wt and NRAS mutated (NRAS-m) [7]. The genetic characterization of these cell lines can be check in the Cancer Cell Line Encyclopedia (https://portals.broadinstitute.org/ccle, access on 1 October 2018).

Three cells lines resistant to vemurafenib were also tested, namely A375-R, SK-MEL-5-R, and 501MEL-R, which were generated from parental cells exposed for 8 weeks to increasing concentrations of PLX4032 (vemurafenib). Similarly to A375 cells, SK-MEL-5 cells and 501MEL are BRAF-m/NRAS-wt and derived from skin metastasis. Resistance to BRAF-I was evidenced by a strong enhancement of the IC_50_ of PLX4032 (10-fold increase) [32].

To investigate the role of apoptosis in the cell death triggered by the NHC compounds, A375 Bcl-xL expressing cells produced as previously described [33].

SK-MEL-28 (ATCC), MeWo (ATCC), HMCB (ATCC), A375-R, SK-MEL-5-R, 501MEL-R cell lines were kindly provided by J. Caramel (CRCL, Lyon, France) [34], the A375 (ATCC) cell line by C. Aspord (Etablissement Français du sang, La Tronche, France) and A375 Bcl-xL by G.Ichim (CRCL, Lyon, France).

All cell lines were tested for mycoplasma with the MycoAlert detection kit (LT07-218-LONZA MycoAlert) every 3 weeks and the number of passages between thawing and use was always under twenty. All cells were cultured at 37 °C in a damp atmosphere containing 5% CO_2_ in DMEM complemented with 10% FBS (Gibco^®^, Thermoscientific Fischer, Villebon-sur-Yvette, France). The BRAF inhibitor resistant cell lines (A375-R, SK-MEL-05-R and 501MEL-R) were cultured in the presence of 3 μmol/L vemurafenib (PLX4032) [35].

#### 4.2.2. MTT Assays

In order to analyze the cytostatic and cytotoxic effects of NHC-Pt-I_2_, NHC-Pt-Br_2_, cisplatin and dacarbazine, as well as to determine their IC_50_ values, cells were seeded onto 96-well plates one day prior to compound treatment with concentrations ranging from 1 to 33.33 μmol/L (or 500 μmol/L for dacarbazine). Treatments were performed for either 1 h (then washed three times and incubated with medium until the viability test) or 72 h. In both cases, the MTT [bromure de 3-(4,5-dimethylthiazol-2-yl)-2,5-diphenyl tetrazolium] test (Promega France, Charbonnières-les-Bains, France) was performed at 72 h.

#### 4.2.3. In Cellulo Pharmacokinetics

To explore the uptake capacities of A375 cells for the 3 Pt compounds, and the kinetics of release of the loaded fraction, cells were sealed in Petri dishes (1 million per dish) for 24-h treatment before being treated for 1 h with 1 μmol/L of each NHC-Pt compound or cisplatin. Cells were washed 3 times and re-cultured. At each time point post-treatment (0 h or 24 h), three cell samples were collected, counted, resuspended in PBS in dedicated perfluoroalkoxy alkane vials, and mineralized with nitric acid to remove all organic compounds before analysis with an ICP-MS instrument (iCAP Q Thermofischer Scientific, Illkirch, France). The mean molar level of total Pt atoms was obtained by dividing the molecular Pt concentration by the total number of cells in each sample. As a control for the ICP-MS method, two samples only containing the treatment solution were also analyzed. Each experiment was repeated 3 to 4 times. The average of the 9 to 12 values obtained for each Pt compound was calculated and compared using a non-parametric *t* test (Mann Whitney—GraphPad Prism, GraphPad Software, Inc, San Diego, CA, USA).

#### 4.2.4. Evaluation of DNA Damage

To evaluate DNA damage triggered by NHC-Pt compounds and cisplatin in A375 cells exposed to these compounds for 1 h at their respective IC_50_ (i.e., 2.5 μmol/L for NHC-Pt-I_2_, 12 μmol/L for NHC-Pt-Br_2_ and 2 μmol/L for cisplatin), the formation of γH2AX foci was qualitatively assessed by optical fluorescence microscopy, immediately after 6 h. To this end, after fixation and permeabilization, cells were incubated at 37 °C (1 h) with the 1:800 diluted mouse γH2AX antibody (Millipore, 05636). Washed cells were then labelled for 20 min at 37 °C with a 1:100 diluted mouse monoclonal anti-FITC antibody (SAB4200738 Sigma-Aldrich, St Quentin Fallavier, France) and then sealed on a slide using DAPI mounting medium (Vectashield^®^ -Abcys, Vector Laboratories, Burlingame, CA, USA).

The cumulative level of γH2AX at 48 h and 72 h post-treatment was quantified by Western blot analysis and compare to the expression of β-Actin used as a normalizing protein. A375 cells treated for 1 h with NHC-Pt compounds or cisplatin at their respective IC_50_ (2.5 μmol/L for NHC-Pt-I_2_, 12 μmol/L for NHC-Pt-Br_2_ and 2 μmol/L for cisplatin) and washed 3 times were then seeded onto 96-well plates, as well as untreated cells (6 samples per condition). At 48 h or 72 h post-treatment, 3 whole samples (i.e., cells and supernatant) per condition were collected. After cell lysis and mixture purification, extracted proteins were labelled with the γH2AX antibody (Millipore 05636, Merck Millipore, Molsheim, France) and ß-Actin antibody (Sigma A5441, Sigma Aldrich) and loaded onto a 12.5% SDS-polyacrylamide gel. The chemiluminescent blots were imaged with the ChemiDoc MP imager (Bio-Rad, Marnes-la-Coquette, France) and background-subtracted density of each band measured with the ImageLab software (Bio-Rad). The ratio of normalized densitometry of γH2AX to ß-Actin was calculated as the protein standardized level of γH2AX expression. A statistical comparison of these values as a function of the post-treatment delay (48 h or 72 h) for each treatment condition was performed using a Sidak’s multiple comparison test (GraphPad Prism™, GraphPad Software, Inc). The mean level of γH2AX expression resulting from treatment with each Pt compound was compared with control untreated cells at 48 h and 72 h using a Dunenett’s multiple comparison test (GraphPad Prism™, GraphPad Software, Inc).

#### 4.2.5. Investigation of Cell Death Processes

To assess the level of cell death resulting from 1-h exposure of A375 cells to the NHC-Pt compounds and cisplatin at their respective IC_50_, we used an IncuCyte^®^ system to monitor in real-time the number of dead cells. To achieve this, cells were cultured post-treatment in a medium containing 0.03 μmol/L of SYTOX-Green™ (Life Technologies, Thermofischer Scientific, Illkirch, France). Three rounds of dead cell counts were conducted every 30 min for 0 h or 83 h, in order to generate the number of dead cells as a function of time for each treatment condition. Each experiment was repeated 3 times. To investigate the involvement of caspases in the cell death process, A375 cells were previously treated with the Pt compounds at their respective IC_50_ (i.e., 2.5 μmol/L for NHC-Pt-I_2_, 12 μmol/L for NHC-Pt-Br_2_ and 2 μmol/L for cisplatin) either in the absence or in the presence of 10 μmol/L QVD (Clinisciences, Nanterre, France) a pan-caspase inhibitor. Cells treated for 1 h with Actinomycin D (a known powerful DNA intercalating agent) at 1 μmol/L were used as a positive control. To investigate the impact of an overexpression of the Bcl-xL protein, implicated in cell resistance to chemotherapy, the importance and the speed of the cell death process was similarly studied using Bcl-xL mutant A375 cells; the overexpression of the anti-apoptotic protein was validated by immunoblot.

### 4.3. Statistical Analysis

All quantitative data are expressed as means and standard deviations of measurements obtained from at least three independent experiments.

## 5. Conclusions

The evaluation of the cytotoxic activities of the two platinum (II) complexes bearing an N-heterocyclic carbene (NHC) ligand on a panel of human metastatic cutaneous melanoma cell lines revealed that NHC-Pt-I_2_ appears to be a promising cytotoxic candidate. It could open research opportunities in the future to improve the prognosis of patients suffering from unresectable metastatic melanoma that are not eligible or that do not respond to the most effective drugs available to date, namely BRAF inhibitors and the anti-PD-1 mAb. In BRAF-m patients, this new type of Pt-based compounds may become a barrier to tumor escape after the onset of resistance to BRAF inhibitors, possibly by extending the delay of response to BRAF inhibitors via the combination of both therapies.

In addition, the conclusions regarding the comparative advantages of the Pt compound NHC-Pt-I_2_ in comparison with cisplatin in terms of cytotoxic efficacy and sensitivity to the resistance mechanism developed by tumor cells, could have a broad impact and potentially be used for other solid cancers.

However, the findings presented in our study should be completed with ex-vivo analyses performed on human tumor samples and pre-clinical studies using relevant mice models including a lymph node and distant metastasis dissemination model and tumor grafting in humanized mice [36], in order to consider the key role of the immune system in the anti-tumor effect in melanoma.

## Figures and Tables

**Table 1:** Compound cytotoxicity induced after 72 h of treatment expressed as mean IC_50_ +/− SD (in μmol/L) according to the genotype of the metastatic cutaneous melanoma cell line.

Compound	A375 ^1^	SK-MEL-28 ^1^	MeWo ^2^	HMCB ^2^
Dacarbazine	9.2 ± 0.2	26.0 ± 16.0	394.0 ± 16.0	>500.0 ^3^
Cisplatin	1.6 ± 0.6	1.0 ± 0.2	13.2 ± 2.4	18.7 ± 4.7
NHC-Pt-I_2_	2.5 ± 0.6	3.1 ± 1.0	4.8 ± 1.4	6.2 ± 3.3
NHC-Pt-Br_2_	12.0 ± 1.0	25.7 ± 4.1	>33.0 ^3^	>33.0 ^3^

^1^ BRAF-m/NRAS-wt cells; ^2^ BRAF-wt/NRAS-m cells; ^3^ undefined IC_50_ values (tested concentrations inducing a cytotoxicity lower than 50%).

**Table 2:** Compound cytotoxicity induced after 1 h of treatment expressed as mean IC_50_ +/− SD (in μmol/L) according to the genotype of the metastatic cutaneous melanoma cell line.

Compound	A375 ^1^	SK-MEL-28 ^1^	MeWo ^2^	HMCB ^2^
Dacarbazine	>33.0 ^3^	>500.0 ^3^	>500.0 ^3^	>500.0 ^3^
Cisplatin	24.6 ± 5.6	>33.0 ^3^	>33.0 ^3^	>33.0 ^3^
NHC-Pt-I_2_	2.3 ± 0.3	5.2 ± 1.7	8.1 ± 0.5	5.4 ± 2.2
NHC-Pt-Br_2_	11.6 ± 0.8	>33.0 ^3^	>33.0 ^3^	>33.0 ^3^

^1^ BRAF-m/NRAS-wt cells; ^2^ BRAF-wt/NRAS-m cells; ^3^ undefined IC_50_ values (tested concentrations inducing a cytotoxicity lower than 50%).

**Table 3:** Compound cytotoxicity induced in BRAF-inhibitor-resistant metastatic cutaneous melanoma cell lines expressed as mean IC_50_ +/− SD (in μmol/L) as a function of treatment duration.

	A375-R ^1^		SK-MEL-5-R ^1^		501MEL-R ^1^	
**Compound**	1 h	72 h	1 h	72 h	1 h	72 h
Dacarbazine	>500.0 ^2^	288.8 ± 182.6	>500.0 ^2^	182.8 ± 138.6	>500.0 ^2^	298.0 ± 68.0
Cisplatin	19.6 ± 9.5	1.0 ± 0.3	14.8 ± 7.4	1.5 ± 0.7	>33.0 ^2^	6.2 ± 2.6
NHC-Pt-I_2_	1.7 ± 0.2	1.2 ± 0.4	1.3 ± 0.3	1.3 ± 0.3	2.5 ± 0.6	2.4 ± 0.5
NHC-Pt-Br_2_	18.0 ± 5.0	16.3 ± 6.9	13.6 ± 2.7	15.3 ± 4.2	25.3 ± 3.2	23.1 ± 2.2

^1^BRAF-m, vemurafenib-resistant cells, ^2^ undefined IC_50_ values (tested concentrations inducing a cytotoxicity lower than 50%).
